# Curcumin-resveratrol nano-formulation counteracting hyperammonemia in rats

**DOI:** 10.1007/s11011-023-01162-1

**Published:** 2023-01-25

**Authors:** Maha Nasr, Omar A.H. Ahmed-farid, Rania F. Ahmed

**Affiliations:** 1 Department of Pharmaceutics and Industrial Pharmacy, Faculty of Pharmacy, Ain Shams University, Cairo, Egypt; 2 Department of Physiology, National Organization for Drug Control and Research, 12553 Giza, Egypt; 3 Department of Pharmacology, Medical Research and Clinical studies Institute, National Research Centre, 12622 Dokki, Giza, Egypt

**Keywords:** Curcumin, Hyperammonemia, Nanoemulsion, Neurotransmitters, Protein deficiency, Resveratrol

## Abstract

Malnutrition and low dietary protein intake could be risk factors for developing peripheral and central hyperammonemia, especially in pediatrics. Both curcumin and resveratrol proved to be effective against several hepatic and cerebral injuries. They were reported to be beneficial in lowering circulating ammonia levels, yet both are known for their low bioavailability. The use of pharmaceutical nano-formulations as delivery systems for these two nutraceuticals could solve the aforementioned problem. Hence, the present study aimed to investigate the valuable outcome of using a combination of curcumin and resveratrol in a nanoemulsion formulation, to counteract protein-deficient diet (PDD)-induced hyperammonemia and the consequent complications in male albino rats. Results revealed that using a nanoemulsion containing both curcumin and resveratrol at a dose of (5 + 5 mg/kg) effectively reduced hepatic and brain ammonia levels, serum ALT and AST levels, hepatic and brain nitric oxide levels, oxidative DNA damage as well as disrupted cellular energy performance. In addition, there was a substantial increase in brain levels of monoamines, and a decrease in glutamate content. Therefore, it can be concluded that the use of combined curcumin and resveratrol nanoemulsion is an effective means of ameliorating the hepatic and cerebral adverse effects resulting from PDD-induced hyperammonemia in rats.

## Introduction

Hyperammonemia is a metabolic condition characterized by elevated ammonia levels, which could lead to the development of acute and chronic liver diseases (Gutiérrez-de-Juan et al. 2017). It is also well established that hyperammonemia resulting from chronic liver disease is a key factor in the pathogenesis of brain dysfunction. Elevated brain ammonia can cause several complications, including impairment of energy utilization by brain cells, as well as disruption of monoaminergic neurotransmitter systems. Studies have demonstrated dopaminergic, noradrenergic and serotonergic neurotransmission alterations by experimentally-induced hyperammonemia (Ronan et al. 2007; Kawai et al. 2012; Higarza et al. 2019; Baraka et al. 2020). In addition, ammonia homeostasis is tightly regulated and linked to the recycling of the major excitatory neurotransmitter glutamate (Cabrera-Pastor et al. 2019), which is the main excitatory neurotransmitter in the central nervous system, and consequently, its alterations are associated with neurological problems (Singh et al. 2014). Particularly, when extracellular glutamate is elevated, glutamate acts as a toxic substance leading to neuronal dysfunction (Ochoa-Sanchez and Rose 2018). Besides, elevated concentrations of ammonia have been shown to generate excessive production of nitric oxide (NOx) (Dasarathy et al. 2017).

Previous investigators have highlighted the hazards of developing hyperammonemia in pediatrics especially in late infancy. Any imbalance in protein and/or energy demands is considered a risk for hyperammonemia (Häberle 2011). Malnutrition, low dietary protein and low amino acids intake can increase peripheral and consequently central ammonia levels (Holecek 2015; Hadjihambi et al. 2018).

Curcumin; a polyphenolic compound, is a component of *Curcuma longa*. It is a well-known anti-inflammatory and antioxidant molecule. Several studies have highlighted the role of curcumin in many different types of human pathologies, including neurocognitive disorders (Di Meo et al. 2019; Yavarpour-Bali et al. 2019). It was also reported to down-regulate the enzymatic activity of inducible nitric oxide synthase (iNOS) (Ghorbani et al. 2014; Boyanapalli and Kong 2015). Besides, curcumin was demonstrated to reduce brain edema after extrahepatic and intrahepatic damage (Frozandeh et al. 2021) as well as lower serum ammonia levels (Huang et al. 2015; Frozandeh et al. 2021).

On the other hand, resveratrol is a natural polyphenolic compound that exhibits beneficial pharmacological properties in a wide range of brain abnormalities; acting mainly as a neuroprotective with monoamines-preserving potential. It was reported to exhibit protective effects against many neurodegenerative diseases (Aguirre et al. 2014; Martínez-Abundis et al. 2016; Ahmed et al. 2020), and it was particularly shown to prevent ammonia toxicity by modulating oxidative stress (Malaguarnera et al. 2018; Kim and Song 2021). Moreover, it was reported that resveratrol exhibited neuroprotective effect against glutamate toxicity (Quincozes-Santos et al. 2014).

Nowadays, therapies based on nanotechnologies have emerged as an innovative and promising alternative to conventional therapy in the treatment of liver diseases (Al-Shakarchi et al. 2018). Therefore, the present study aimed to investigate the beneficial effects of using a combination of resveratrol and curcumin in a nanoemulsion formulation, to counteract protein-deficient diet (PDD)-induced hyperammonemia, and the consequent development of brain dysfunction in juvenile male albino rats.

## Materials and methods

### Solvents and drugs

Curcumin, resveratrol, phenobarbital, sodium hydrogen phosphate, potassium dihydrogen phosphate, methanol, acetonitrile, potassium hydroxide, ammonium acetate, potassium phosphate and sodium chloride were purchased from Sigma Aldrich, Germany. Labrafac Lipophile WL 1349 was kindly gifted by Gattefosse’, France. Cremophor RH was kindly gifted by BASF, Germany.

### Preparation and characterization of nanoemulsions

Preparation of the nanoemulsions of curcumin, resveratrol and their combination was carried out using the spontaneous emulsification method as previously described (Nasr 2016). Briefly, 50 mg of each drug or both drugs were dissolved in Labrafac Lipophile oil (10% w/v) and Cremophor RH surfactant (10% w/v), followed by their gradual addition to a magnetically-stirred aqueous phase at room temperature (IKA C-MAG HS 7, Germany). The nanoemulsions were characterized for their particle size, polydispersity and surface charge using the Zetasizer device (Malvern, UK), after dilution 1:100 with deionized water at 25 °C, and equilibration time of two minutes. The detection angle was 173 °C, and the refractive index was set to 1.33 (Nasr 2016).

### Animals

Male Wistar albino juvenile rats weighing 60–70 g were purchased from the National Research Centre (NRC) animal house. Animals were acclimatized for seven days before proceeding further with the experimental research. All experimental procedures followed the regulations of the Research Ethics Committee of the National Research Centre (approval number 2,415,062,022) in accordance with relevant national guidelines and regulations and compliance with the ARRIVE guidelines.

### Experimental design

Rats were divided into eight groups (n = 16). The first group was designated as the normal control and was fed a normal standard recommended rats’ pellet diet for seventy-five days, kept in the same room and under the same conditions as all the other groups, and ingested 5 ml/kg distilled water daily during the last fifteen days. The seven other groups were fed a protein-deficient diet (PDD) consisting of pellets of shelled corn grains for 75 days (Ahmed-Farid et al. 2017). Drug treatments started from day 61 and for 15 days; in which two groups (RL and RH) ingested the resveratrol nanoemulsion at two dose levels (2.5 and 5 mg/kg, p.o), two groups (CL and CH) ingested the curcumin nanoemulsion at two dose levels (2.5 and 5 mg/ kg, p.o), two groups (MRCL and MRCH) ingested the combination nanoemulsion at two dose levels (2.5 + 2.5 and 5 + 5 mg/ kg, p.o). The final group assigned as PDD control was given daily oral ingestion of distilled water (5 ml/ kg, p.o).

On day 76, after the last treatments ingestions by 24 h, rats in each group were divided randomly into two subsets of 8 rats; the first subset was subjected to phenobarbital anesthesia for blood samples collection, and the other subset was sacrificed by decapitation for brain and liver tissues’ collection. Tissues were kept at -80 °C for further studies.

### Quantification of serum ALT and AST levels (IU/L) in serum

Serum total aspartate aminotransferase (AST) and alanine aminotransferase (ALT) levels were measured spectrophotometrically with JASCO V-630 spectrophotometer (Serial No. C322461148 Japan) using a kit provided by a local supplier (Bio-diagnostic Co.) (Reitman and Frankel 1957). For more information, the kit’s manual is provided as supplementary material.

### Quantification of hepatic and brain ammonia (µmol/g), NOx (µmol/g), 8-OHdG content (pg/g), ATP, ADP and AMP (µg/g), brain 5-HT, NE, DA and glutamate (µg/g) in brain or liver tissues

Brain or liver tissues were homogenized in phosphate buffer. The supernatant was separated. Ammonia was assessed spectrophotometrically with JASCO V-630 spectrophotometer (Serial No. C322461148 Japan), in which 1 ml of liver or brain supernatant was added to 1.5 ml of phenol–nitroprusside reagent and 2 ml of sodium hypochlorite reagent and the mixture was left for 20 min at room temperature. The color intensity was then measured at 630 nm against distilled water blank, and ammonium chloride (0.1–1.0 mmol) was used as standard (Oyovwi et al. 2021).

For all other brain and liver parameters, HPLC system (Agilent HP 1200 series, USA) consisting of quaternary pump, column oven, Rheodine injector and 20 µl loop, and UV variable wavelength detector was used. The report and chromatograms were taken from Chemstation program (Agilent, USA). For the assessment of brain glutamate level, C18 reversed phase column was used. The mobile phase was a seven-step gradient of increasing concentrations of solvent B from 5 to 70%. Solvent A was composed of 50 mM ammonium acetate (pH 6.5) and solvent B was 100 mM ammonium acetate (pH 6.5): acetonitrile (1:1). The flow rate was 2 ml/min, and detection was performed at wavelength 254 nm. (Ahmed-Farid et al. 2017). For serotonin (5-HT), norepinephrine (NE) and dopamine (DA) levels, an AQUA column C18 was used. The mobile phase was 20mM potassium phosphate, pH 2.5: methanol (99:1), the flow rate 1.5ml/min, and detection was done at wavelength 210 nm (Pagel et al. 2000). For the brain and hepatic nitric oxide (nitrates + nitrites) (NOx) levels, the analytical column was anion exchange PRP-X100 Hamilton. The mobile phase was a mixture of 0.1 M sodium chloride:methanol, at a volume ratio of 45:55, the flow rate was 2 ml/min, and the detection wavelength was 230 nm (Papadoyannis et al. 1999). For the brain and hepatic 8-hydroxy-2-deoxyguanosine (8-OHdG) levels, the analytical column was C18 reverse phase column. The eluting solution was water:methanol at a ratio of 85:15 with 50 mM potassium dihydrogen phosphate pH 5.5, at a flow rate of 0.68 ml/min; detected at wavelength 245 nm (Lodovici et al. 1997). For the brain and hepatic adenosine tri, di and monophosphate (ATP, ADP and AMP) levels, the analytical column was Ultrasphere ODS EC column. The mobile phase A consisted of potassium dihydrogen phosphate solution adjusted to pH 7.0 using 0.1 mol/L potassium hydroxide, while mobile phase B consisted of 100% acetonitrile. Flow rate of the mobile phase was 1.2 ml/min, and the detection wavelength was 254 nm. The resulting data was also used for computation of AMP/ATP ratio as well as the total adenylate energy charge (AEC) according to the equation: (ATP + 1/2ADP)/(ATP + ADP + AMP) (Atkinson and Walton 1967; Teerlink et al. 1993; Saleh et al. 2017b).

### Statistical analyses

Statistical analyses were carried out using one-way ANOVA followed by Tukey’s multiple comparisons test. P < 0.05 was accepted as significant in all types of statistical tests. Graph prism software, version 9 was used to carry out all statistical tests. Values were expressed as means ± S.E. Pearson’s correlation study was conducted using the same software program where the difference was considered significant at P < 0.0001.

## Results

### Characterization of the nanoemulsions

The properties of the prepared nanoemulsions are displayed in Table 1.

**Table 1:** Particle size, polydispersity index and zeta potential of the prepared nanoemulsions

	Particle size (nm)	Polydispersity index	Zeta potential (mV)
**Curcumin nanoemulsion**	136 ± 2.96	0.31 ± 0.01	-17.89 ± 2.3
**Resveratrol nanoemulsion**	128 ± 5.78	0.34 ± 0.06	-15.92 ± 3.7
**Curcumin-Resveratrol nanoemulsion**	135 ± 2.05	0.39 ± 0.03	-18.93 ± 2.8

### Impact on serum hepatic enzymes ALT and AST

**Fig. 1:**
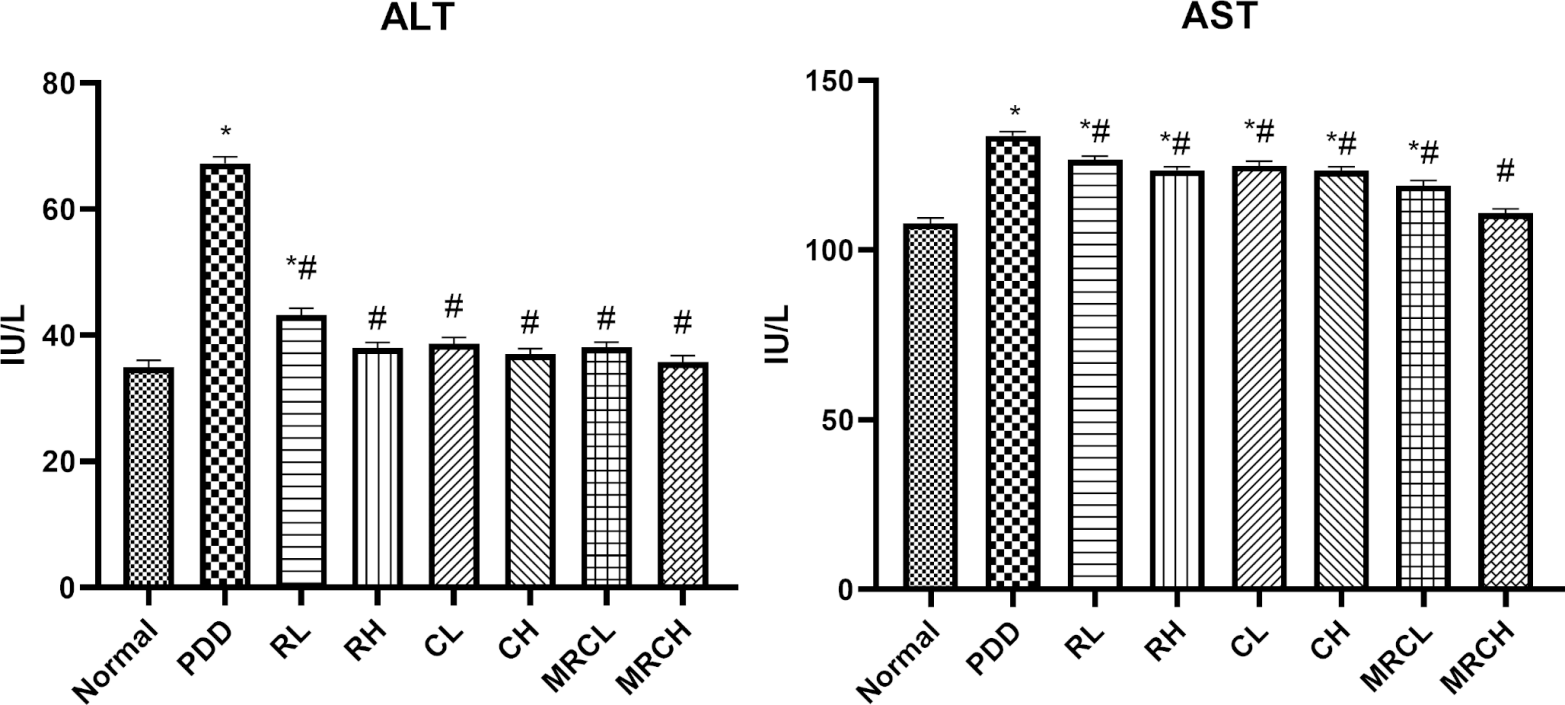
Impact of different treatments on serum hepatic enzymes ALT and AST. ^*^ Significance vs. normal, ^#^ significance vs. PDD control

Both ALT and AST levels were significantly elevated by 92.61% and 23.82% respectively, after chronic ingestion of the PDD diet for seventy-five days as compared to the normal group. Generally, most treatment groups normalized ALT levels and significantly reduced AST levels compared to the PDD control group. It is worth mentioning that ingesting the nanoemulsion formulation containing both curcumin and resveratrol at a dose level of 5 + 5 mg/kg normalized both ALT and AST levels **(**Fig. 1**)**.

### Impact on hepatic and brain ammonia levels

**Fig. 2:**
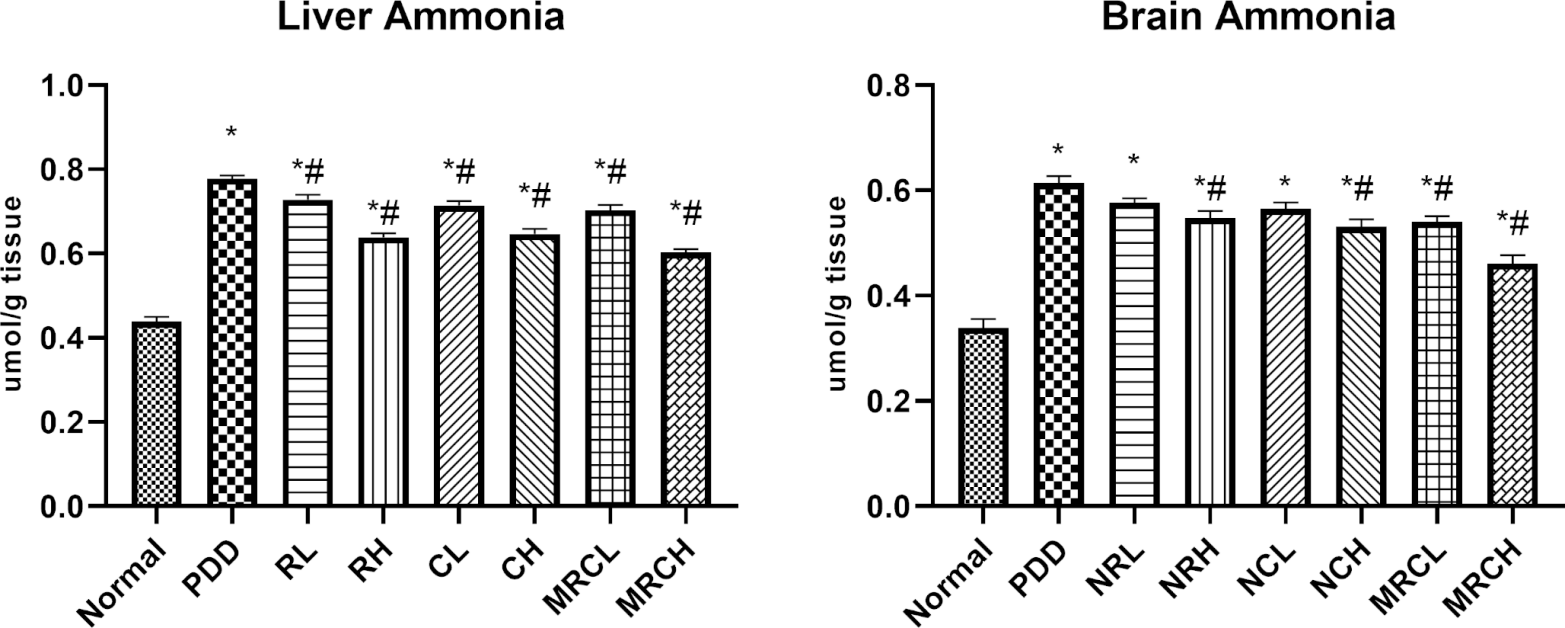
Impact of different treatments on hepatic and brain ammonia levels. ^*^ Significance vs. normal, ^#^ significance vs. PDD control

Chronic ingestion of the PDD diet resulted in a significant elevation in the hepatic ammonia level, and consequently the brain ammonia level by 77.19% and 81.17% respectively, compared to the normal group. All nanoformulations significantly reduced the hepatic ammonia levels, whereas; only curcumin and resveratrol ingested at the higher dose level (5 mg/kg) as well as both dose levels of the combined formulations significantly reduced the brain ammonia level **(**Fig. 2**)**.

### Impact on hepatic and brain NOx levels and 8-OHdG levels

**Fig. 3:**
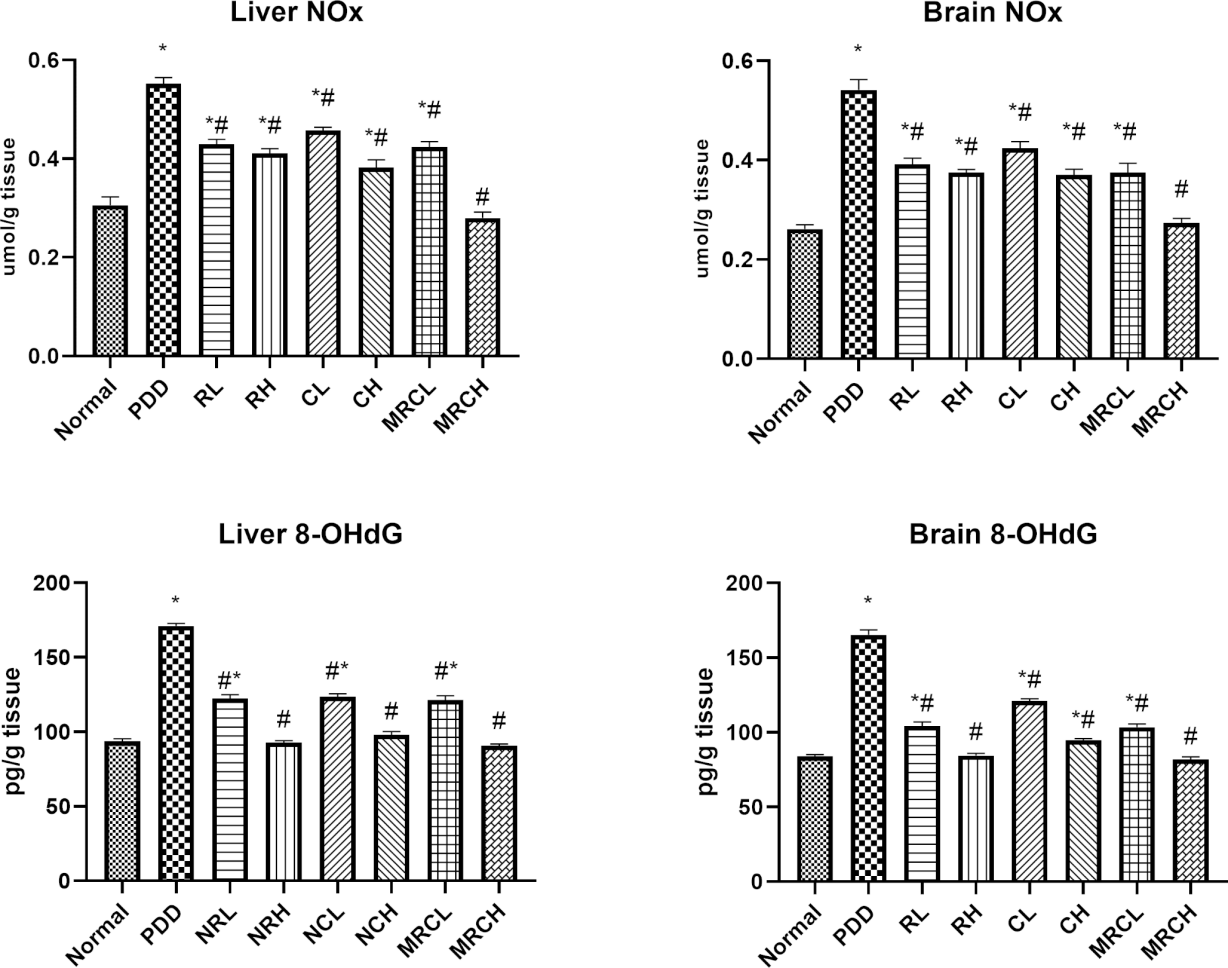
Impact of different treatments on hepatic and brain NOx and 8-OHdG levels. ^*^ Significance vs. normal, ^#^ significance vs. PDD control

The protein-deficient diet resulted in an extensive elevation in both hepatic and brain NOx levels by 80.79% and 107.16% respectively, as well as a substantial increase in the 8-OHdG levels by 82.4% and 96.97% respectively, as compared to the normal group. Ingesting the combined formulation at the higher dose level normalized both parameters in both organs. In addition, all other treatment groups significantly reduced both parameters compared to the PDD group **(**Fig. 3**).**

### Impact on hepatic and brain cellular energy parameters

**Fig. 4:**
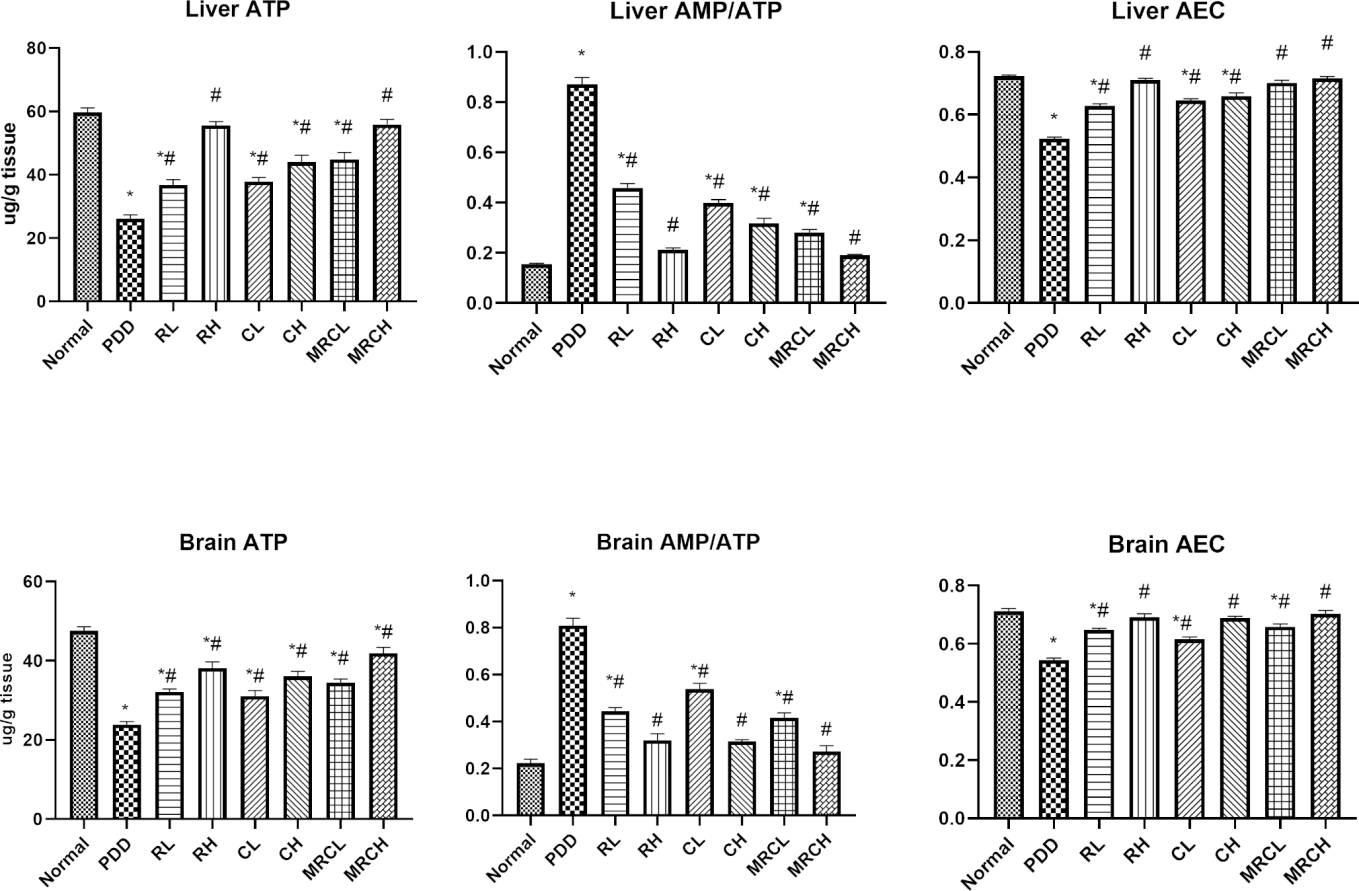
Impact of different treatments on hepatic and brain cellular energy parameters. ^*^ Significance vs. normal, ^#^ significance vs. PDD control

Ingestion of PDD significantly disrupted the hepatic and brain cellular energy parameters, represented by a substantial decrease in the ATP levels by 56.3% and 49.8% respectively, as well as a significant decrease in the AEC ratios (0.52, 0.54 vs. 0.72, 0.71) respectively, in addition to extensive increase in the AMP/ATP ratios (0.87, 0.81 vs. 0.16, 0.22) respectively, as compared to the normal group. All treatment groups managed to ameliorate this disruption in cellular energy parameters compared to the PDD group, where the most promising results were obtained when ingesting the combination formulation of the higher dose levels of MRCH **(**Fig. 4**)**.

### Impact on brain monoaminergic neurotransmitters and glutamate levels

**Fig. 5:**
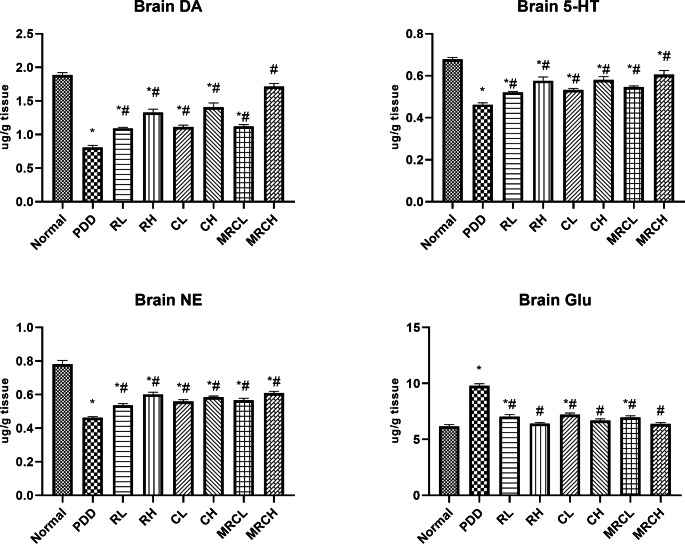
Impact of different treatments on brain monoaminergic neurotransmitters and glutamate levels. ^*^ Significance vs. normal, ^#^ significance vs. PDD control

Ingestion of PDD chronically resulted in a significant reduction in serotonin, dopamine, and norepinephrine levels by 31.86%, 57.32% and 40.8% respectively, compared to the normal group. Meanwhile, it substantially elevated the glutamate level by 58.6%. Treatment with the formulations under investigation significantly ameliorated the disruption in the monoaminergic neurotransmitters and glutamate levels (Fig. 5).

### Correlation studies

**Fig. 6:**
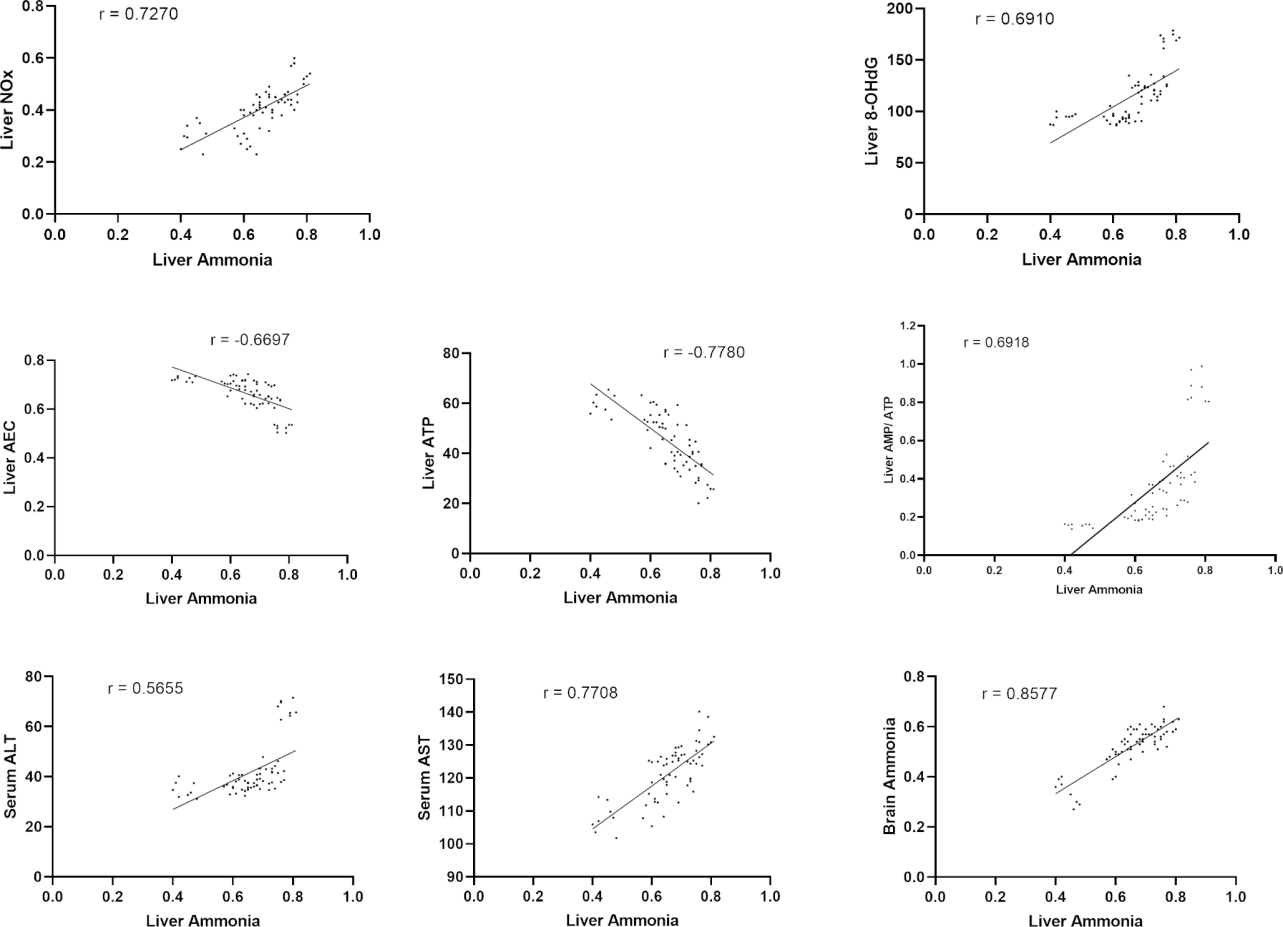
Correlation of different parameters to hepatic ammonia level at p < 0.0001

**Fig. 7:**
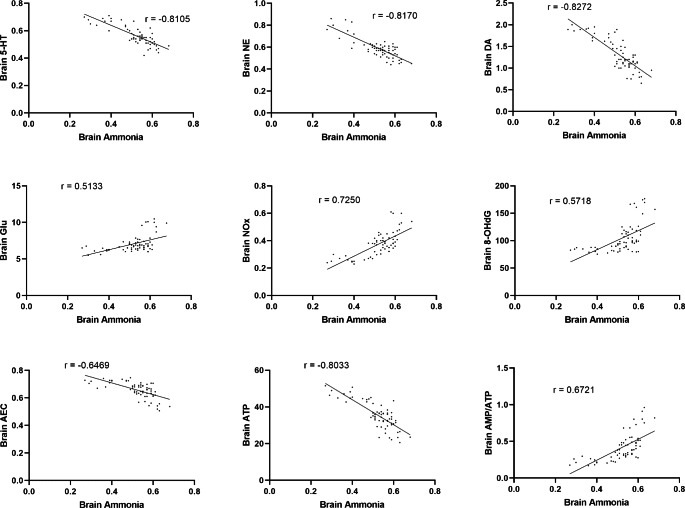
Correlation of different parameters to brain ammonia level at p < 0.0001

Correlation studies revealed a positive correlation between the elevated hepatic ammonia level and hepatic levels of NOx, 8-OHdG, and AMP/ATP ratio, as well as serum ALT and AST. There was also a negative correlation between elevated hepatic ammonia level and hepatic ATP level as well as hepatic AEC ratio, indicating that elevated hepatic ammonia level is a direct cause of liver injury represented by the pronounced nitrosative stress, oxidative DNA damage, disrupted cellular energy and elevated serum hepatic enzymes levels. Moreover, there was a positive correlation between elevated hepatic ammonia and elevated brain ammonia levels. Besides, elevated brain ammonia level was directly correlated to elevated brain contents of glutamate, NOx, 8-OHdG, AMP/ATP ratio, and inversely correlated to brain levels of monoamines, ATP, and AEC ratio; designating that brain hyperammonemia could be a leading cause of development of cerebral dysfunction. **(**Figs. 6 and 7**)**

## Discussion

The present study aimed to investigate the beneficial effects of using resveratrol and curcumin in nanoemulsions either alone or in combination to counteract the adverse effects of ingesting a PDD in juvenile rats chronically for seventy-five days, represented by the development of hepatic and brain hyperammonemia and the accompanying consequences on brain monoaminergic and glutamatergic systems.

Hyperammonemia resulting from liver damage is considered a major risk factor for the development of brain abnormalities due to alteration of monoaminergic and glutamatergic neurotransmitter systems, overproduction of nitric oxide (NOx), oxidative DNA damage, impaired ATP generation, accompanied by a disruption in cellular energy (Albrecht and Zielińska 2019; Yan et al. 2021; Hadjihambi et al. 2022). Ammonia is a weak base in the body consisting of two molecular forms, NH_3_ and NH_4_^+^, that can readily cross the Blood-brain barrier (BBB) (Vairappan et al. 2019). In hyperammonemia, the over influx of ammonia from the blood to the brain occurs, where it accumulates and acts as a neurotoxin due to prompting a series of neurological abnormalities including bioenergetic failure, oxidative/nitrosative stress, glutamate-mediated neurological death, and cerebral edema (Dasarathy et al. 2017; Oyovwi et al. 2021). In addition, elevated ammonia levels have long been proven to be associated with depletion in serotonin, norepinephrine and dopamine (Albrecht and Wegrzynowicz 2005; Higarza et al. 2019; Baraka et al. 2020).

Dietary protein deficiency and decreased body intake of essential amino acids, especially the branched-chain amino acids (BCAAs), were previously reported to be associated with severe liver damage associated with a disrupted capacity to detoxify ammonia through the urea cycle and consequent elevation in ammonia levels (Katayama 2020). Besides, it was formerly stated that ingestion of a choline-deficient diet resulted in a significant increase in ammonia levels with a high degree of hepatic steatosis and inflammation (Gutiérrez-de-Juan et al. 2017). Similarly, several investigations highlighted that chronic ingestion of methionine-choline deficient diet resulted in a significant elevation in serum ALT and AST along with pronounced hepatic oxidative stress and DNA fragmentation as well as elevation in brain ammonia and NOx levels (Tahan et al. 2009; Amin et al. 2017; Saleh et al. 2017a). In particular, this condition can be very hazardous in pediatrics suffering from malnutrition, hence inadequate protein and amino acids supplementation could eventually lead to the development of hyperammonemia (Häberle 2011; Holecek 2015; Hadjihambi et al. 2018).

Our results revealed that PDD ingestion by juvenile rats led to pronounced status of hyperammonemia both in the liver and brain, along with elevation in the nitrosative stress and oxidative DNA damage, in addition to definite disturbance in the maintenance of cellular energy balance. Serum liver enzymes ALT and AST were increased, indicating hepatic malfunctioning. In addition, serotonin, norepinephrine and dopamine levels were reduced, while the glutamate level was elevated, indicating the possibility of the development of central neuronal excitotoxicity. Correlation studies revealed the existence of a positive correlation between elevated hepatic ammonia levels and brain ammonia levels. Besides, there was a direct correlation between elevated hepatic ammonia levels as well as brain ammonia levels and the elevation in NOx levels, the existence of oxidative DNA damage, and disruption in cellular energy parameters in the corresponding organ. Hepatic ammonia level was also found to correlate with the elevated serum ALT and AST. Furthermore, elevated brain ammonia level was inversely correlated to the brain levels of monoamines. In addition, it could be estimated that PDD-induced hyperammonemia would lead to brain excitotoxicity, as a positive correlation between brain ammonia level and brain glutamate level was detected. Thus, hyperammonemia resulting from PDD would be considered a hazardous risk factor for hepatic as well as cerebral damages.

Current strategies in the management of hyperammonemia aim to decrease ammonia levels and thus target the modulation of the metabolic processes and organs involved in ammonia detoxification, in addition to the down-regulation of oxidative stress (Germoush et al. 2018). Curcumin and resveratrol are two polyphenols that have been extensively studied in the medicinal field as nutraceuticals with documented antioxidant and anti-inflammatory effects, resulting in well-established hepato- and neuro-protective potentials (Malaguarnera et al. 2018; Moore et al. 2018; Khan et al. 2019; Galiniak et al. 2019; Ahmed et al. 2020; Benameur et al. 2021; Miguel et al. 2021; Jabczyk et al. 2021; Chupradit et al. 2022). Unfortunately, both resveratrol and curcumin exhibit low bioavailability and solubility (Liu et al. 2016; Galiniak et al. 2019; Ma et al. 2019; Jabczyk et al. 2021). Pharmaceutical nanotechnology has proven to provide superior drug delivery systems using several techniques, to encapsulate both nutraceuticals in nanoformulations that showed promising results in the management of several hepatic complications as well as neurodegenerative disorders due to enhanced bioavailability (Ganesan et al. 2015; Vasconcelos et al. 2019; Moradi et al. 2020; Grilc et al. 2021; Elbaset et al. 2022).

In the present study, curcumin and resveratrol nanoemulsions significantly ameliorated the adverse effects of PDD-induced hepatic and brain hyperammonemia in juvenile rats, indicating antioxidant potential as well as pronounced hepatoprotective and neuroprotective effects. The high dose level combination of MRCH (5 + 5 mg/kg) exhibited the most promising results regarding the reduction in serum ALT and AST levels, declination in hepatic and brain ammonia levels, reversal of hepatic nitrosative stress as well as oxidative DNA damage, elevation in brain monoamines levels, along with the decrease in glutamate level, and improvement of hepatic and brain cellular energy indices.

Our study is in agreement with previous investigations in which the hepato- and neuroprotective effects of curcumin, either when used in conventional form or nanoformulations, have been extensively investigated, and were generally attributed to antioxidant, anti-inflammatory and DNA preservation potentials (Farzaei et al. 2018; Kheiripour et al. 2021; Nebrisi 2021; Moghaddam et al. 2021). Curcumin consumption in several models of drug-induced hepatotoxicity in rats resulted in decreased serum levels of hepatic enzymes including ALT and AST, declined serum ammonia levels, and hindrance of hepatic oxidative stress (Khan et al. 2019; Frozandeh et al. 2021). Moreover, it was reported that curcumin consumption could ameliorate nicotine-induced oxidative stress in the liver, kidney, spleen and lungs in female rats under protein-restricted diet (Maiti et al. 2015). Similarly, curcumin was found effective in counteracting methionine-choline deficient diet-induced elevation in serum ALT as well as increased hepatic oxidative stress and 8-OHdG, which is a well-known marker of oxidative DNA damage (Leclercq et al. 2004; Vizzutti et al. 2010). In addition, the previous investigation in our laboratory highlighted the beneficial effects of curcumin nanoemulsion in counteracting high-fat, high-fructose (HFHF)-induced hepatic damage represented by the reduction of the elevated serum ALT, AST levels and hepatic MDA, NOx, 8-OHdG levels along with elevation of the declined hepatic GSH level and AEC ratio (Elbaset et al. 2022). Meanwhile, curcumin was also reported to be effective in counteracting cerebral ischemia-induced oxidative stress and glutamate excitotoxicity (Subedi and Gaire 2021) and proved to be effective in inhibiting the expression of monoamine oxidase (MAO-A and MAO-B) enzymes leading to increased levels of norepinephrine, serotonin, and dopamine (Bhat et al. 2019; Matias et al. 2021). In addition, curcumin iron oxide nanoparticles were reported to reduce brain NOx levels and elevate serotonin and dopamine levels in rats’ reserpine model of depression (Khadrawy et al. 2021).

On the other hand, resveratrol exhibited pronounced antioxidant potential in several models, principally *via* the control of major antioxidant enzymes, and block of DNA damage by free radicals (Pal and Sarkar 2014; Szkudelski and Szkudelska 2018; Meng et al. 2021). Former investigations demonstrated that it could also decrease the level of 8-OHdG, suppress NOx production, and reverse the disruption of energy homeostasis by elevating the production of ATP in cellular mitochondria (Saleh et al. 2017b; Ahmed et al. 2020; Meng et al. 2021; Kim and Song 2021). Furthermore, *in-vitro* and *in-vivo* studies delineated the ability of resveratrol to improve liver functions represented by declination in ALT and AST levels, reduction of ammonia levels, maintenance of the blood-brain barrier (BBB) integrity, amelioration of hyperammonemia-induced mitochondrial dysfunction, and disruption in the glutamatergic system and cellular redox imbalance (Bobermin et al. 2015, 2018; Vairappan et al. 2019). Besides, resveratrol was previously reported to partially prevent low protein diet-induced maternal as well as offspring oxidative stress and metabolic dysfunction (Vega et al. 2016). Similarly, it was demonstrated to reduce serum ALT, AST as well as hepatic MDA, and ameliorate methionine-choline deficient diet-induced hepatic steatosis in mice (Ji et al. 2015; Kong et al. 2022). The neuroprotective effect of resveratrol could be attributed to improved liver function and, thus, ammonia metabolism, as well as direct antioxidant and anti-inflammatory potentials both on the liver and brain tissues (Vairappan et al. 2019). In addition, resveratrol was previously reported as a neurotransmitter enhancer (Sarubbo et al. 2015; Gu et al. 2019; Ahmed et al. 2020).

Regarding nanoformulations of resveratrol, it was formerly reported that resveratrol selenium nanoparticles exhibited ameliorative effects against aluminium chloride-induced cerebral dysfunction and cognitive defects and the action was attributed to the antioxidant and anti-inflammatory potentials (Abozaid et al. 2022). Besides, nanostructured lipid carriers containing resveratrol ameliorated oxidative stress and cerebral inflammation following ischemic stroke in rats (Ashafaq et al. 2021). Moreover, resveratrol nanoemulsion prepared by spontaneous emulsification method, followed by high-pressure homogenization using vitamin E:sefsol (1:1) as the oil phase was reported to counteract haloperidol-induced neuronal damage and oxidative stress in brain tissue (Pangeni et al. 2014).

## Conclusion

To our knowledge, this is the first report of using a combined resveratrol and curcumin nanoemulsion to counteract PDD-induced hyperammonemia in juvenile rats, and according to the results, it can be delineated that the nanoemulsion was effective in ameliorating both the hepatic and cerebral adverse effects. A limitation of the current study is the absence of tissue histological examinations, which would have provided more insights on the nanoemulsion’s therapeutic effect. Futuristic work will also include the quantification of brain cell death using annexin V assay. Moreover, further investigations are necessary to estimate the effectiveness of this strategy in managing such complications in pediatrics suffering from malnutrition-induced hyperammonemia.

## Data Availability

The datasets generated during and/or analyzed during the current study are available from the corresponding author on reasonable request.

## Abbreviations

ATP: Adenosine triphosphate
ADP: Adenosine diphosphate
AMP: Adenosine monophosphate
AEC: Adenylate energy charge
ALT: Alanine aminotransferase
AST: Aspartate aminotransferase
BBB: Blood-brain barrier
DA: Dopamine levels
GSH: Glutathione
MDA: Malondialdehyde
NOx: Nitric oxide
iNOS: Nitric oxide synthase
NE: Norepinephrine
PDD: Protein-deficient diet
5-HT: Serotonin
8-OHdG: 8-hydroxy-2-deoxyguanosine
